# Editorial: Taking a Fresh Look at Old Zoonoses, What Have We Been Missing in One Health Research and Education?

**DOI:** 10.3389/fpubh.2022.895277

**Published:** 2022-04-25

**Authors:** Alessandra Scagliarini, Olli Peltoniemi, Anita Luise Michel

**Affiliations:** ^1^Department of Experimental, Diagnostic and Specialty Medicine, University of Bologna, Bologna, Italy; ^2^Department of Production Animal Medicine, University of Helsinki, Helsinki, Finland; ^3^Department of Veterinary Tropical Diseases, University of Pretoria, Pretoria, South Africa

**Keywords:** One Health (OH), neglected zoonotic diseases (NZDs), zoonosis, emerging infectious disease (EIDs), education, research

In this Research Topic, we present a collection of papers focusing on the role of One Health education and research to promote and protect the health of human, animals, and environment. Covid-19 is the most recent demonstration of a human health crash in the post vaccine era, the pandemic has reminded that human health is inextricably linked to that of animals and the environment. Boriani et al. discussed the planetary aspects of the pandemic, and proposed a method aimed at easing the approach to systems and inter- and trans-disciplinary thinking to find nature-based solutions (NbS) for preventing future pandemics. A reclassification of Covid-19, as an emerging infectious disease (EID) was suggested by Haider et al. Withdrawing the designation of SARS-CoV-2 as a zoonosis and reviewing COVID-19 as an EID will be important to address the underlying drivers of the emergence of such pathogens and reduce the risk of inappropriate animal persecution or other unsuitable interventions. Looking at COVID-19 as an EID, makes it no less valuable that research confirms whether an animal reservoir actually exists becoming potential sources of future human infection. Emerging zoonoses are a rising threat to global health, having caused severe economic impacts in the past years. However, the greatest burden on human health and livelihoods is caused by endemic zoonoses that are persistent health problems around the world. This Research Topic particularly focuses on “neglected” zoonotic diseases, affecting poor, and marginalized populations in low-resource settings. For these diseases, whose burden has proven difficult to estimate, it is still virtually impossible to assess the real impact on the social wellbeing and the mental health of affected communities, livestock owners, and their families (1). Tuberculosis and Brucellosis are important chronic infections that are endemic in many parts of the world, especially in developing countries (2). Endemic diseases cannot be readily eradicated but best be monitored and acted upon with a control plan in the animal reservoir if and when the threshold of concern is reached. Therefore, surveillance plans play a crucial role in keeping neglected zoonoses at bay (3) as suggested by some authors in this Research Topic.

Angkwanish et al. focused on wildlife, reporting the seroprevalence *M. tuberculosis* (MTBC) in captive and wild Asian elephants (*Elephas maximus*) in Thailand. The Authors underlined the importance of surveillance and control programs to reduce the risk of transmission amongst elephants and from elephants to humans and other species. Similarly, Jamil et al. discuss the importance of epidemiologic surveillance for the assessment of the role of wildlife and other species to serve as reservoirs of Brucellosis, a persisting health hazard in Pakistan. The Authors underlined the importance of education on “One Health” concepts to secure the farmers' acceptance of the control measures implemented and, with reference to Food safety regulations, also consumers education should be strengthened to reduce the risks of foodborne transmission.

Developing initiatives and education programs is crucial to better respond to existing and emerging environmental and epidemiological crises. Butala et al. conveyed that, despite being often overlooked and underused in favor of more complex solutions, education is a key tool for the control of Neglected Zoonotic Diseases (NZDs). The Authors presented the successes and benefits of several education initiatives as a control tool for NSDs, in low and middle income countries. Successful education programs, targeted to children of school age, carried out in Tanzania, Kenia, and China created long lasting impressions on children with a great potential to reduce subsequent number of cases of NZDs through the adoption of changes in the environment and active involvement of the communities in sanitation practices.

The importance of education, to reduce the zoonotic risks, was also promoted by Zucca et al. The Authors presented the results of a survey carried out among adolescents in six different countries (Italy, Austria, Slovenia, Germany, Mauritius, and Japan) indicating that the circularity of the One Health concept, related to the transmission of diseases from animals to humans and vice-versa, is not well-understood by a large proportion of the respondents. The potential of theoretical and practical lessons in the classroom were shown to improve the basic understanding on the transmission of zoonotic diseases, underlying the importance of teaching health prevention with a One Health approach in school curricula. The role of University is crucial and many are already engaged in developing initiatives and education programs at different levels and orientation toward One Health in research is increasing. Aragrande et al. argued that One Health is more than a declared concept and it is rarely assessed. In fact, to effectively address the complex problems related to Health, it is therefore necessary to put an inter and transdisciplinary approach including human and veterinary medicine, traditional public health, food safety, with the involvement of other disciplines including social sciences and humanities, but this is not always applied. To evaluate the research processes and working habits of researcher teams, under the OH lens, the OH-ness degree should be estimated. The Authors implemented a methodology developed by the EU-COST Action, “Network for the Evaluation of One Health (NEOH) in 2018 (4).” A first application of this methodology was attempted to evaluate the One Health-ness of one of the academic team participating in the KA2 Capacity Building project “ELEPHANT” (Empowering universities' Learning and rEsearch caPacities in the one Health Approach for the maNagement of animals at the wildlife, livestock and human interface in SouTh Africa). The project aims at embedding OH in research and learning to enable the control of diseases at the human, animal, and environmental interface. An increased level of OHness, at the end of the project, is considered a positive quality indicator.

As guest editors, we believe that this Research Topic's contributions and conclusions will assist readers in expanding their current knowledge. Furthermore, this should inspire them to put in place further collaborations across disciplines to lay the foundation for future education and research initiatives. These multidisciplinary collaborative efforts should promote one health approach, to the address the most important future health challenges with the best science-based control measures.

## Author Contributions

All authors listed have made a substantial, direct, and intellectual contribution to the work and approved it for publication.

## Conflict of Interest

The authors declare that the research was conducted in the absence of any commercial or financial relationships that could be construed as a potential conflict of interest.

## Publisher's Note

All claims expressed in this article are solely those of the authors and do not necessarily represent those of their affiliated organizations, or those of the publisher, the editors and the reviewers. Any product that may be evaluated in this article, or claim that may be made by its manufacturer, is not guaranteed or endorsed by the publisher.
